# Supplementary material for: Chemogenetics defines a short-chain fatty acid receptor gut–brain axis
Source: eLife. 2022 Mar 1;11:e73777. doi: 10.7554/eLife.73777 (PMC8887895; doi:10.7554/eLife.73777)
Supplement: Supplementary file 1. — n cells isolated from DRGs from N hFFA2-DREADD-HA-expressing mice were assessed for capacity to elevate intracellular [Ca2+] in response to MOMBA or MOMBA following the indicated treatments. See also Figure 4B. [file elife-73777-supp1.docx]

**Supplementary file 1: MOMBA promotes Ca^2+^ elevation in subsets of DRG-derived cells in a FFA2 and G_q_/G_11_-dependent manner**

|  | **MOMBA** | **MOMBA + CATPB** | **MOMBA +**  **FR900359** | **MOMBA +**  **P.toxin** |
| --- | --- | --- | --- | --- |
| Animals (N) | 9 | 7 | 5 | 6 |
| Cells (n) | 103 | 68 | 63 | 74 |
| % activated  Mean +/- S.E.M. | 41.4 +/- 10.0 | 2.6 +/- 1.7 | 3.4 +/- 1.9 | 39.2 +/- 4.9 |
