# Supplementary material for: Chemogenetics defines a short-chain fatty acid receptor gut–brain axis
Source: eLife. 2022 Mar 1;11:e73777. doi: 10.7554/eLife.73777 (PMC8887895; doi:10.7554/eLife.73777)
Supplement: Supplementary file 2. — n cells isolated from dorsal root ganglia (DRGs) from N hFFA2-DREADD-HA-expressing mice were assessed for capacity to elevate intracellular [Ca2+] in response to C3 or C3 following the indicated treatments. See Figure 4C for details. [file elife-73777-supp2.docx]

**Supplementary file 2: C3 promotes Ca^2+^ elevation in subsets of DRG-derived cells in a non-FFA2 and G_i_-dependent manner**

|  | **C3** | **C3 + CATPB** | **C3 +**  **FR900359** | **C3 +**  **P.toxin** |
| --- | --- | --- | --- | --- |
| Animals (N) | 7 | 5 | 4 | 5 |
| Cells (n) | 84 | 41 | 52 | 59 |
| % activated  Mean +/- S.E.M. | 81.1 +/- 3.8 | 73.0 +/ -6.6 | 82.5 +/- 4.3 | 11.0 +/- 5.6 |
