# Supplementary material for: Chemogenetics defines a short-chain fatty acid receptor gut–brain axis
Source: eLife. 2022 Mar 1;11:e73777. doi: 10.7554/eLife.73777 (PMC8887895; doi:10.7554/eLife.73777)
Supplement: Supplementary file 3. [file elife-73777-supp3.docx]

**Supplementary file 3: Activation of gut SCFA receptors promotes c-Fos expression in the dorsal horn of the spinal cord**

|  | **Saline (N=4)** | **C3 (N=4)** | **Saline (N=7)** | **MOMBA (N=7)** |
| --- | --- | --- | --- | --- |
| Animals | Wild type | Wild type | hFFA2-DREADD-HA | hFFA2-DREADD-HA |
| c-Fos expressing cells (per square mm) | 27.7 +/- 6.9 | 54.0 +/- 3.9 | 26.0 +/- 1.6 | 40.5 +/- 3.0 |

See **Figure 5** for further details.
